# Hospitalization of injured pregnant women: a decade of data

**DOI:** 10.1186/s13584-025-00727-y

**Published:** 2025-11-12

**Authors:** Sharon Goldman, Morel Ragoler, Adi Givon, Irina Radomislensky, Eldad Katorza, Israel Trauma Group, H. Bahouth, M. Bala, A. Bar, A. Braslavsky, D. Czeiger, D. Fadeev, A. L. Goldstein, I. Grevtsev, E. Hashavia, G. Hirschhorn, I. Jeroukhimov, A. Kedar, Y. Klein, A. Korin, B. Levit, U. Neeman, I. Schrier, A. D. Schwarz, W. Shomar, O. Yaslowitz, I. Zoarets

**Affiliations:** 1 The National Center for Trauma and Emergency Medicine Research, Gertner Institute for Epidemiology and Health Policy Research, Sheba Medical Center, Ramat Gan, Israel; 2 School of Medicine, Tel Aviv University, Tel Aviv, Israel; 3 Gertner Institute for Epidemiology & Health policy Research, Chaim Sheba Medical Center, Ramat Gan, Israel; 4 Antenatal Diagnostic Unit, Department of Obstetrics and Gynecology, Chaim Sheba Medical Center, Ramat Gan, Israel; 5 Arrow Program for Medical Research Education, Chaim Sheba Medical Center, Ramat Gan, Israel

**Keywords:** Hospitalization, Pregnant women, Trauma-related injuries, Gestational age, Traffic collisions, Falls

## Abstract

**Background:**

Pregnant women commonly sustain injuries following traffic collisions, falls, and intentional incidents such as domestic violence. Injuries sustained by pregnant women can lead to placental abruption, pelvic fracture, preterm delivery as well as maternal and fetal mortality. The aim of this study was to compare injury and hospitalization characteristics among hospitalized pregnant and nonpregnant women. For pregnant women, gestational age was analyzed according to injury severity and hospitalization characteristics.

**Methods:**

The Israel National Trauma Registry was the data source for this retrospective study. Demographic, injury and hospitalization characteristics were collected and analyzed for pregnant and nonpregnant women hospitalized between Jan 1, 2012 and December 31, 2021. Among pregnant females, gestational age was identified. Categorical variables were compared using the Chi-square Test and Fisher’s Exact Test.

**Results:**

A total of 33,377 women, aged 18–45 years, were hospitalized due to trauma-related injury; 14,606 (43.8%) were pregnant, and 18,771 (56.2%) were not pregnant. Among the pregnant women, 91.7% had an Injury Severity Score (ISS) of 1, and 75.9% were hospitalized for a single day. In comparison, 31% of the nonpregnant women had an ISS of 1 (*P* < 0.0001), and 32% were hospitalized for one day. Traffic accidents contributed to 51.8% of hospitalizations among pregnant women, compared with 42.8% among nonpregnant women. While falls were more prevalent among pregnant women, a greater proportion of nonpregnant women were hospitalized with intentional injuries. Among pregnant women, injuries during the third trimester are most common. However, those hospitalized during the first trimester suffered from more severe injuries than injuries during the second and third trimesters did. Compared with nonpregnant women, pregnant women are more likely to sustain minor injuries, have shorter hospitalization stays, have fewer surgical interventions and have fewer admissions to intensive care units (ICUs).

**Conclusions:**

This study provides important data for medical personnel and policymakers regarding trauma-related injuries among pregnant women. The results highlight the need to reassess observation and treatment protocols to balance appropriate maternal and fetal care while minimizing unnecessary hospitalizations. Collaboration among policymakers, obstetricians, and neonatal specialists is essential to refining evidence-based protocols that improve outcomes for pregnant trauma patients and their fetuses.

## Background

Trauma-related injuries during pregnancy have significant implications for both maternal and fetal health [1–3]. Injury outcomes among pregnant women include placental abruption, pelvic fracture, preterm delivery and elevated mortality rates for both the mother and fetus [3–5]. 

Pregnant women commonly sustain injuries following traffic collisions, falls, and intentional incidents such as domestic violence [2, 6, 7]. Traffic collisions are the leading cause,, with a 2019 literature review reporting mortality rates of 13.7% for pregnant women and 10.7% for fetuses [2–4, 8]. Falls, affecting 25% of pregnant women, often result from increased body weight and shifts in weight distribution and balance [8]. 

Managing trauma in pregnancy presents medical challenges due to physiological changes, necessitating a multidisciplinary team that includes obstetric and trauma specialists.(2,34,9,10) P. ()The risk of significant fetal injuries and even mortality among injured pregnant women emphasizes the importance of treatment protocols and policies [3]. 

The Advanced Trauma Life Support (ATLS) guidelines recommend a pregnancy test for all women of reproductive age in trauma care [2, 10]. Initial treatment should follow trauma protocols [2, 8, 10], including assessing uterine displacement, evaluating vital signs and performing actions to stabilize the pregnant patient following an injury event (e.g., maternal hemodynamic stability via ABCDE (airway, breathing, circulation, disability, and exposure) [3, 4, 6, 8, 10]. 

Fetal monitoring should be begin once maternal stability is ensured. Current guidelines recommend variable durations of fetal monitoring following trauma. Minor injuries typically require at least 4–6 h of observation, while severe cases (including vaginal bleeding or positive Kleihauer–Betke (KB) tests) necessitate 24 h.In viable pregnancies (> 22 weeks) up to 48 h of observations may be indicated. These recommendations underscore the need to tailor monitoring based o maternal injury severity and gestational age, balancing maternal and fetal safety with the avoidance of unnecessary hospitalization [1, 3, 6, 8, 10]. 

While observation is essential for maternal and fetal health, unnecessary hospitalizations for pregnant women increase the risk of hospital acquired infections, anxiety and emotional distress for the pregnant women as well as pose a financial burden on the individual, institution and health system due to do unwarranted expenses. Developing efficient and effective evidence-based treatment protocols for pregnancy is crucial for improving maternal and fetal outcomes following trauma.

This study aims to analyze and characterize the demographic, injury, and hospitalization patterns among pregnant and nonpregnant women hospitalized due to trauma-related injuries. Additionally, it seeks to examine injury characteristics among pregnant women in relation to gestational age to enhance clinical management protocols for pregnant trauma patients. Lastly, this study will compare hospitalization patterns between pregnant and nonpregnant women to identify potential cases of unjustified hospitalizations.

## Methods

### Study design

The Israel National Trauma Registry (INTR) was the data source for this retrospective study. The INTR includes comprehensive data on hospitalized trauma patients from 21 hospitals, of which six are level I trauma centers (TCs) and 14 are level II TCs. Trained trauma registrars recorded data from each trauma center under the guidance of a trauma director. Data quality checks were conducted before data analysis. The data are anonymous. All hospitalized trauma patients classified with an ICD-9-CM diagnosis code 800–989.9 who were admitted to the Department of Emergency Medicine (ER) and hospitalized, died in the ER, or were transferred to another hospital were included in the database. The INTR does not include casualties who died onsite or en route to the hospital, admissions 72 h or more after the incident, poisonings, suffocations, or drownings. This study was approved by the Sheba Medical Center Institutional Review Board (IRB) (SMC 5138–18).

For the purposes of this study, the data obtained from the INTR included pregnant (cases) and nonpregnant (control) women hospitalized following a trauma-related incident between January 1, 2012, and December 31, 2021. All women aged 18–45 years were included in this study. A negligible percentage of pregnant women under the age of 18 (0.32%) and older than 45 years (0.22%) were also considered during this process. The comparison with nonpregnant women serves to conceptualize hospitalization patterns and injury severity but is not intended to suggest equivalency in clinical decision-making criteria.

Data regarding gestational age at the time of trauma-related incidents were also included in this study. Injured pregnant women and medical staff reported pregnancy status and related information. Age, ethnicity, length of stay, injury severity, and injured body region were analyzed in relation to gestational age. Trimester classification was only applied to the subset of pregnant women who reported gestational age.

The variables extracted from the INTR included:

*Demographic information*: Age and ethnicity (Jew/non-Jew).

*Mechanism of injury*: traffic crash (private vehicle, pedestrian, bus, bicycle, motorcycle, and other), fall (from height, same level, and stairs), burns, intentional (violence, suicide, terror/war), and other unintentional injuries. (Suicide due to strangulation and poisoning is not included in the INTR).

*Seat position for two- and four-wheel vehicles* (driver, front seat, rear seat).

*Type of injury*: blunt, penetrating.

*Injury Severity*: The Injury Severity Score (ISS), is the sum of the squares of the single highest AIS score for each of the three most severely injured body regions.

*Injured body region*: head/neck, torso, extremities; pelvic fractures.

*Hospital resource utilization: Length of stay (LOS),* admission to intensive care units (ICUs), and surgical intervention.

*Evacuation Method:* private vehicles and emergency medical services (EMS).

*Gestational age:* The length of time that a fetus grows inside the mother’s uterus, in weeks. The gestational age was divided into trimesters.

### Statistical analysis

Categorical variables were compared using the chi-square test or Fisher's exact test depending on the sample size of the groups. A t- test was used to investigate the normally distributed age among the investigated groups. The median age is presented as the interquartile range (IQR). All the statistical analyses were performed using S.A.S. software; version 9.4 (SAS Institute, Cary, NC, USA). All comparisons were considered statistically significant at P<0.05.

## Results

### Characteristics of the study population

**Fig. 1:**
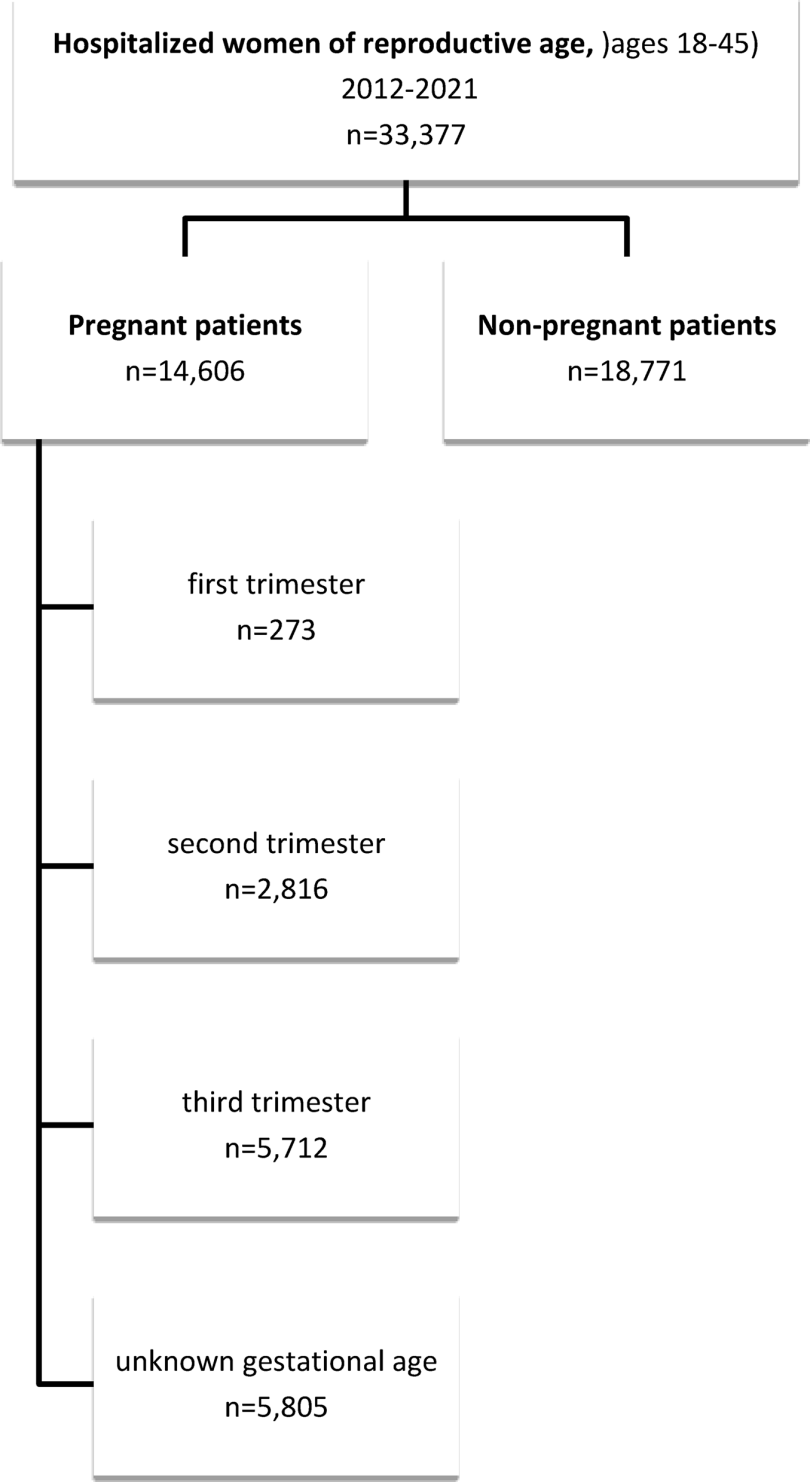
Study population- women ages 18–45 hospitalized with injuries, 2012–2021. The flowchart presents the final study population from the total number hospitalized adult women with trauma injuries and included in the Israel National Trauma Registry between 2012 and 2021

A total of 33,377 women aged 18–45 years, were hospitalized between 2012 and 2021 due to trauma-related injuries. Among them, 14,606 (43.8%) were pregnant at the time of hospitalization, while 18,771 (56.2%) were not pregnant (Fig. 1).

Among the pregnant women, only 8,801 (60.3%) reported their gestational age. Of the 14,606 pregnant women, 5,712 (39.1%) sustained injuries during the third trimester, 2,816 (19.3%) during the second trimester, and 273 (1.9%) during the first trimester; gestational age was unknown for 5,805 (39.7%) patients (Fig. 1).

Pregnant women were generally younger that their nonpregnant counterparts, with a median age of 29.0 year (IQR 25.0,33.0) compared to 30.0 years (IQR 23.0, 38.0) among nonpregnant women (*P* < 0.0001). Additionally, non-Jewish individuals comprised 24% of the pregnant cohort and 32.8% of nonpregnant cohort. (*P* < 0.0001) (Table 1).

### Injury mechanism

Traffic collisions (51.8% vs. 42.8%) and falls (40.3% vs. 31.0%) were the primary causes of hospitalization for pregnant and nonpregnant women, respectively (*P* < 0.0001) (Table 1).

Among pregnant women hospitalized due to traffic-related injuries, 90.2% (*n* = 6,818) were injured in private vehicles (*P* < 0.0001) and 67% (*n* = 5,069) were drivers (*P* < 0.0001). In comparison, 56.1% of nonpregnant women hospitalized with traffic-related injuries were injured in private vehicles. Nonpregnant women were more likely injured as pedestrians (16.3%) compared to pregnant women (3.2%). (Table 1) Seat position data indicated that 67% of pregnant women and 52.4% of nonpregnant women were driving during the traffic collision (data not shown).

Falls accounted for 40% (*n* = 5,888) of hospitalizations among the pregnant women, of which 72.2% (*n* = 4,52) were same-level falls, 5.7% (*n* = 337) were from height, and 20.4% (*n* = 1,199) were on stairs. In contrast, falls comprised 31% of hospitalizations among nonpregnant women, of which 59.7% were at the same level, 19.2% were from heights and 18.9% were on stairs (*P* < 0.0001) (Table 1).

Intentional injuries were more common in nonpregnant women (6.5%, *n* = 1,217) than pregnant women (2.3%, *n* = 328). (Table 1) Among intentional injuries, violence was the cause in 90.2% of the pregnant patients and 58.6% of the nonpregnant patients. Eleven pregnant women were hospitalized for attempted suicide, whereas among nonpregnant women, 31.3% (*n* = 381) of intentional injuries were suicide attempts (*P* < 0.0001) (Table 1).

The method of arriving at the trauma center was reported, which included EMS or private cars. Pregnant casualties were more likely to be transported to the hospital by private cars (76.6%), whereas nonpregnant women were more likely to arrive by EMS (50.9%) (*P* < 0.0001) (Table 1).

### Hospital resource utilization

A significant proportion of pregnant women (75.9%) were hospitalized for a single day, compared to 32.0% of nonpregnant women (*P* < 0.0001) (Table 2). Nonpregnant women were more likely than pregnant women to be admitted to the ICU (5.9% and 0.4% for nonpregnant and pregnant women, respectively) (*P* < 0.0001). Similarly, surgical interventions were more common among nonpregnant women (37.6%) compared to pregnant women (2.3%)(*P* < 0.0001) (Table 2).

## Injury severity

The majority of hospitalized pregnant women had an ISS of 1 (91.7%, *n* = 13,385), whereas only 31% (*n* = 5,802) of the nonpregnant women had an ISS of 1 (*P* < 0.0001) (Table 2). Analysis of hospitalization and injury characteristics for women with an ISS *≥* 2, revealed consistent findings regarding LOS (*P* < 0.0001), ICU admissions (*P* < 0.0001), and surgical interventions (*P* < 0.0001) (Table 2).

Among pregnant women, the torso was the most commonly affected body region (77.0%), whereas among nonpregnant women, extremity injuries were predominant (61.2%). Only 13.9% of pregnant women sustained extremity injuries (*P* < 0.0001). Head and neck injuries were reported in 6.9% (*n* = 1,001) of pregnant women and 30.6% (*n* = 5,743) of nonpregnant women(*P* < 0.0001) (Table 1). The majority of injuries among pregnant women were classified as blunt trauma 99.3% (*n* = 14,504) of all injuries. In-hospital mortality was rare, three pregnant patients (0.02%) and 115 (0.6%) nonpregnant patients succumbed to their injuries. All three pregnant women suffered from severe head injuries and an ISS *≥* 25. Likewise, the majority (76%) of nonpregnant women who died suffered from head injuries and 90% had an ISS *≥* 25.

Among patients with an ISS *≥* 2 57% of pregnant women sustained torso injuries compared to 29% of nonpregnant women (*P* < 0.0001). Head and neck injuries occurred in 20.3% of pregnant women and 29.1% of nonpregnant women (*P* < 0.0001) with no significant differences in extremity injuries (*p* = 0.79). (Data not shown)

**Fig. 2:**
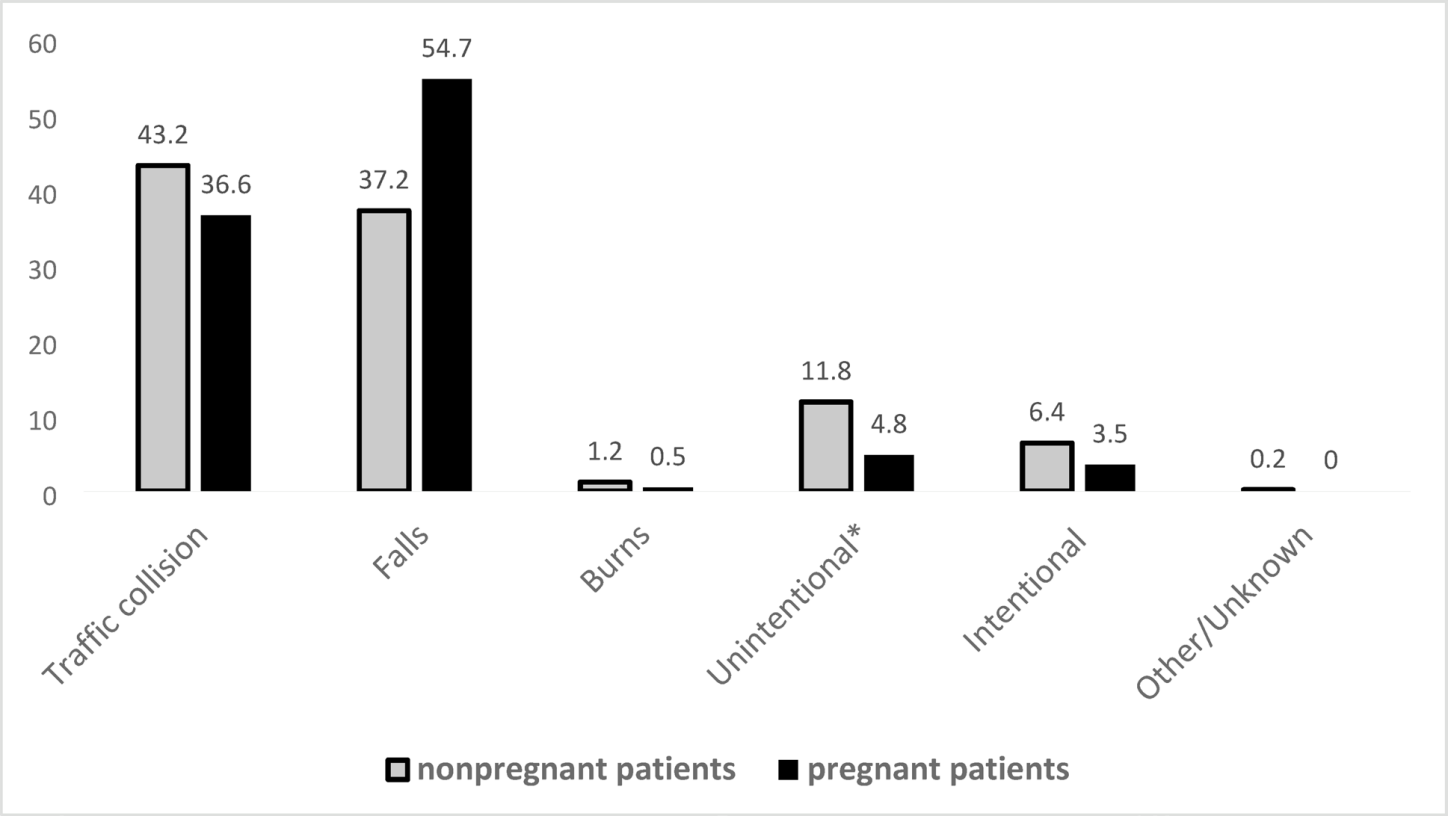
Injury mechanism of hospitalized pregnant and nonpregnant women (ISS *≥* 2), 2012–2021. Figure 2 presents the distribution of hospitalized pregnant and nonpregnant women, with an ISS *≥* 2, by injury mechanism. As shown, pregnant women were more likely to endure a fall related injuries, whereas nonpregnant women were more likely to be hospitalized for traffic and intentional injuries. Other/unknown refers to mechanism of injury not classified in the above groups or unknown mechanism of injury (*n* = 23,0.18%) for nonpregnant women

Among casualties with ISS *≥* 2, falls accounted for 54.7% (*n* = 666) of hospitalizations among pregnant women and 37.2% (*n* = 4,809) among nonpregnant women. Traffic collisions resulted in hospitalizations in 36.6% (*n* = 445) and 43.2% (*n* = 5,586) of pregnant and nonpregnant women with ISS *≥* 2, respectively *P* < 0.0001) (Fig. 2).

## Gestational age

Among pregnant women, 8,801 (60.3%) reported their gestational age; of which, 5,712 (64.9%) were injured during the third trimester, 2,816 (32%) during the second trimester, and 273 (3.1%) during the first trimester. More than half of the hospitalizations during the first (54.2%) and second (55.7%) trimesters were due to traffic collisions. Among women hospitalized during the third trimester, 46.6% were due to traffic collisions and 46.3% were fall injuries. Intentional injuries were more prominent among women in their first (9.2%) trimester than in their second (3.4%) and third (1.4%) trimesters (*P* < 0.0001) (Table 3).

Hospitalizations for more than one day were more common during their first trimester (46.5%) than during the second (23.1%) and third (23.7%) trimesters (*P* < 0.0001) (Table 3). A greater proportion of women with an ISS *≥* 2 were hospitalized during the first trimester (36.0%) than during the second (9.4%) and third trimesters (8.4%) (*P* < 0.0001) (Table 3). Injuries to the torso were more frequent in the second (81.4%) and third (77.6%) trimesters than in the first trimester (64.5%) (*P* < 0.0001). Conversely, head and neck injuries (28.2%) and extremity injuries (37.0%) were more prevalent during the first trimester (*P* < 0.0001) (Table 3).

## Discussion

Data from the INTR provide extensive insight into trauma-related hospitalizations. This study focused on pregnant and nonpregnant women aged 18–45 years who were hospitalized over a ten-year period. The findings indicate that pregnant women were more likely than nonpregnant women to sustain minor injuries, whereas nonpregnant women had longer hospital stays and higher rates of surgical interventions. It should be noted that the comparison between pregnant and nonpregnant patients is intended to identify differential patterns that may guide resource allocation and policy not to evaluate clinical appropriateness per se.

Notably, most hospitalized pregnant women were injured during the third trimester, followed by the second trimester (for patients with known gestational age). However, women in the first trimester tended to sustain more severe injuries and required longer hospitalizations. The findings of this study reported higher injury severity (ISS > 2) among pregnant patients in the first trimester, which may have contributed to longer LOS in this group. During the first trimester there is increased concern for early pregnancy loss which may result in more monitoring during early gestation.

In this study, the majority of pregnant women sustained minor injuries and were hospitalized for only one day. A possible explanation, as suggested by Azar et al., is that pregnant women are more likely than nonpregnant women to seek medical care following a traumatic event [15]. Additionally, concerns regarding fetal well-being often lead to hospitalization even in cases of minor injuries [14, 15]. Low injury severity among hospitalized pregnant women has been documented in previous studies [7, 9, 14–18]. This is reflected by the relatively low incidence of medical interventions, such as surgery and blood transfusions, in this population [7, 9, 15]. 

Between 2012 and 2021, traffic collisions were the leading cause of hospitalizations among women aged 18–45, the majority of whom were drivers in private cars. Consistent with previous research, traffic-related events were the primary cause of injury among pregnant women [7, 9, 11–14]. However, the proportion of pregnant women hospitalized due to traffic collisions varied across studies. In the present study, 51.8% of the pregnant women were hospitalized due to a traffic collision, whereas studies from other countries reported results ranging from 55.5% in the UK, to 82.5% in Australia [7, 9, 11–13]. Additionally, approximately 90% of pregnant women hospitalized due to traffic-related injuries were injured while in a private vehicle, and the majority being drivers. The high incidence of torso injuries in this group may be attributed to the proximity of the abdomen to the steering wheel, a finding supported by previous research indicating that 88.6%−90% of pregnant women sustain injuries in private vehicles [14, 15]. Studies in Israel and the U.S. similarly reported that 67% and 54% of hospitalized pregnant women involved in motor vehicle accidents were drivers, respectively [14, 15]. Thoracic and abdominal injuries were the most common injuries reported [15], Likewise, 14.6% of pregnant Japanese women sustained chest and abdominal injuries, respectively, during a 15-year period [9]. The variation between our findings (51.8% of admissions due to traffic collisions) and higher rates reported in the UK and Australia may reflect differences in healthcare systems, medico-legal environments, and safety culture. In many settings, obstetricians adopt a more cautious approach to avoid potential litigation related to adverse fetal outcomes. In contrast, Israel currently lacks dedicated national protocols for pregnant trauma patients, resulting in heterogeneity of practice and a tendency toward conservative admission policies. Our findings highlight the need for national guidelines to ensure consistent, evidence-based management.

In contrast to previous research, the current study observed a higher percentage of hospitalizations due to falls among pregnant women. Falls accounted for approximately 40% of hospitalizations in this study, compared to 9.5%−16.7% in other studies [7, 11, 12]. Differences in study design and population characteristics may explain this variation. For example, this study included all hospitalized pregnant women, whereas Sato et al. focused on younger women with severe trauma (ISS > 12) [11]. The discrepancies in the study periods may have also contributed to the observed differences. Longer study periods may capture more variation in seasonal trends, societal behaviors, and data recording practices. These factors could explain the higher fall rates observed compared to prior studies with shorter timeframes.

Similar to the current study, Deshpande et al. examined a ten-year period, whereas other studies covered between two- and twelve-year periods [11, 12]. 

A short LOS for hospitalized pregnant women has been well- documented [9, 14–17]. For example, in Australia, 78% of pregnant women were hospitalized for a single day, and only 12% of pregnant and 28% of nonpregnant women involved in traffic collisions were hospitalized for six days or more [16]. However, a study conducted at a single level I trauma center in the U.S. found no significant differences in LOS, mortality, ICU, or surgery rates between pregnant and nonpregnant women involved in traffic accidents [18]. The relatively low percentage (2.3%) of surgical interventions among pregnant women in this study differs from the 12.1% reported in a U.S. single level I trauma center study [17]. These discrepancies may be attributed to differences in data sources, as the current study analyzed data from multiple trauma centers rather than a single center. It should be emphasized that the INTR has standardized data collection protocols and trained registrars to ensure high data quality.

The findings of the current study also indicate that most hospitalized pregnant women with known gestational age were injured during the third trimester, which is also supported by previous research [5, 12]. For example, between 2000 and 2008 approximately 70% of hospitalized injured pregnant women in Israel (sustained injuries during the third trimester [5]. A slightly lower percentage (47.7%) was reported in Qatar between 2013 and 2015 [12]. As shown in this study, most pregnant women hospitalized due to falls were injured during the second and third trimesters, likely due to changes in body weight and the center of gravity during these stages of pregnancy [4]. The reduced postural stability in the third trimester increases susceptibility to falls [2]. 

Fetal monitoring is often conducted due to concerns regarding placental abruption, a potential consequence of trauma [6, 19]. However, the monitoring duration varies and should be case-specific. While minor injuries are not associated with adverse pregnancy outcomes, severe trauma may lead to placental abruption, preterm birth or fetal and maternal mortality [6, 18]. Thus, distinguishing between necessary and unwarranted hospitalizations is crucial.

Unnecessary hospitalizations impose burdens on the pregnant woman, her family and the healthcare system. The hospital environment can cause anxiety especially for pregnant woman, and unwarranted hospitalization (even if brief) and justified longer stays for more severe injuries may result in financial strain due to lost workdays and childcare responsibilities. While brief stays may seem minimal, even short admissions can lead to anxiety and unnecessary costs if not medically indicated. While in Israel pregnant trauma patients are most often admitted to high risk obstetric wards, where infection risk is generally lower than in surgical or trauma wards, and where staff are trained in maternal-fetal monitoring. Nonetheless, hospitalization is not without consequences. Even brief admissions may cause emotional distress, disruption of family life, and unwarranted healthcare costs. These considerations reinforce the importance of evidence-based admissions criteria to minimize avoidable hospitalizations while ensuring patient safety..

### Limitations

A key limitation of this study is that the INTR lacks comprehensive obstetric data, including fetal health and outcomes. Consequently, fetal injuries and outcomes due to the trauma-related injuries were not analyzed. Additionally, the INTR includes only hospitalized patients, excluding those discharged from the emergency department without hospitalization. However, this limitation does not compromise the study’s objective, which was focused on examining hospitalized patients.

### Policy implications and recommendations

Careful assessment of both maternal and fetal health is essential when managing injured pregnant women in the emergency department. This study highlights the need for clear, evidence-based protocols to guide observation and treatment. Although trauma is the leading cause of non-obstetric maternal mortality, most hospitalized pregnant women sustain minor injuries. While some trauma cases may result in adverse fetal outcomes, unnecessary hospitalizations should be minimized to optimize resource allocation and patient care [6, 18]. 

Updated guidelines should integrate outpatient care and monitoring strategies to reduce unwarranted hospitalization stays, with hospitalization decisions based on maternal injury severity and gestational age [8, 10]. Since the risk of fetal loss is higher in the first two trimesters and the third trimester is associated with increased risks of fetal distress, premature birth, and stillbirth, gestational age should be a key factor in determining monitor duration. Given that fetal compromise usually becomes apparent within the first 4–6 h of electronic monitoring, pregnant women with minor injuries should be observed for at least six hours [20, 21]. If no complications arise, patients should be discharged for outpatient follow-up rather than prolonged hospitalization.

In this study, a substantial proportion of hospitalized women had an ISS of 1, many of whom could have been monitored in the ED for six hours and discharged with appropriate outpatient care. While patients with an ISS of 1 are generally suitable for outpatient monitoring, those with an ISS > 8 should remain hospitalized for continued maternal and fetal observation. Discharge criteria should include stable maternal vital signs (normal blood pressure, pulse, and respiratory rate), absence of vaginal bleeding or uterine contractions, normal fetal heart rate, and no need for additional medical interventions [21]. 

To establish comprehensive guidelines, a multidisciplinary panel of experts, including obstetricians, neonatologists, and trauma specialists, should develop standardized hospitalization and discharge criteria for injured pregnant women. These protocols should not only be implemented in the hospital settings, but also be extended to community healthcare providers to ensure effective and optimal trauma care for pregnant patients.

Current international guidelines differ in their recommendations. For example, the American College of Obstetricians and Gynecologists (ACOG) advises a minimum of 4–6 h of continuous fetal monitoring after maternal stabilization, extending to 24 h in cases of major trauma, uterine tenderness, or vaginal bleeding. The Canadian guidelines similarly emphasize monitoring tailored to injury severity and gestational age. At present, the Israeli Society of Obstetrics and Gynecology has not published specific national guidelines on trauma during pregnancy, and practice generally follows ATLS and obstetric principles. These gaps underscore the importance of developing standardized, locally relevant protocols in Israel.

## Conclusion

Pregnant women are more likely than nonpregnant women to be hospitalized after sustaining minor injuries, raising questions about the necessity of such admissions. This study highlights the need to reassess observation and treatment protocols to balance appropriate maternal and fetal care while minimizing unnecessary hospitalizations. Updating guidelines can help ensure optimal trauma management, prioritizing both safety and efficacy. Collaboration among policymakers, obstetricians, and neonatal specializes is essential to refining evidence-based protocols that improve outcomes for pregnant trauma patients and their fetuses.

### Statistical analysis

Categorical variables were compared using the chi-square test or Fisher’s exact test depending on the sample size of the groups. A t- test was used to investigate the normally distributed age among the investigated groups. The median age is presented as the interquartile range (IQR). All the statistical analyses were performed using S.A.S. software; version 9.4 (SAS Institute, Cary, NC, USA). All comparisons were considered statistically significant at *P* < 0.05. In Table 1, subheadings and sub group totals are marked in bold. 

**Table 1:** Demographic and injury characteristics among hospitalized pregnant and nonpregnant women, 2012–2021

	Pregnant women *n* (%)	Nonpregnant women *n* (%)	*P*-value
**Total**	14,606	18,771	
**Age** Median (IQR 25,75)	29.0 (25.0,33.0)	30.0 (23.0, 38.0)	
Mean (SD)	29.3 (± 5.3)	30.8 (± 8.5)	
**Ethnicity n (%)** ^1^			< 0.0001
Jews	11,071 (76.1)	12,425 (67.2)	
Non-Jews	3,483 (23.9)	6,064 (32.8)	
**Injury Mechanism**			< 0.0001
Traffic	7,563 (51.8)	8,039 (42.8)	
Falls	5,888 (40.3)	5,811 (31.0)	
Intentional	328 (2.3)	1,217 (6.5)	
Other unintentional^2^	807 (5.6)	3,704 (19.7)	
**Traffic**,** of which**:	**7**,**563**	**8**,**039**	< 0.0001
Private vehicle	6,818 (90.2)	4,506 (56.1)	
Pedestrian	243 (3.2)	1,312 (16.3)	
Bus	188 (2.5)	143 (1.8)	
Bicycle	17 (0.2)	397 (4.9)	
Motorcycle	16 (0.2)	551 (6.9)	
Others/unknown	281 (3.7)	1,130 (14.1)	
**Falls**, **of which**:	**5**,**888**	**5**,**811**	< 0.0001
Fall on same level	4,252 (72.2)	3,468 (59.7)	
Fall on stairs	1,199 (20.4)	1,096 (18.9)	
Fall from height	337 (5.7)	1,116 (19.1)	
Other fall	100 (1.7)	131 (2.3)	
**Intentional**,** of which**:	**328**	**1**,**217**	< 0.0001
Violence	296 (90.2)	713 (58.6)	
Suicide attempts	11 (3.4)	381 (31.3)	
Terror/warfare	21 (6.4)	123 (10.1)	
**Injured body region** ^3^			
Head and Neck	1,001 (6.9)	5,743 (30.6)	< 0.0001
Torso	11,250 (77.0)	6,087 (32.4)	< 0.0001
Spine/back	192 (1.3)	2,139 (11.4)	< 0.0001
Extremities	2,031 (13.9)	11,491 (61.22)	< 0.0001
**Method of transportation**			< 0.0001
Private vehicle	11,194 (76.6)	9,213 (49.1)	
Emergency medical services	3,412 (23.4)	9,558 (80.9)	

P<0.0001

^1^Unknown ethnicity, n=334

^2^Other unintentional includes burns, other and unknown unintentional injuries

^3^Casualties can sustain injuries to more than one body region

**Table 2:** Hospitalization and injury severity characteristics among hospitalized pregnant and non-pregnant women, 2012–2021

Injury Severity Characteristics	ALL hospitalized women		Hospitalized women classified with ISS *≥* 2	
	Pregnant women *n* (%) (*n* = 14,606)	Non-pregnant women n (%) (*n* = 18,771)	Pregnant women *n* (%) (*n* = 1,217)	Nonpregnant women *n* (%) (*n* = 12,928)
**LOS (days)**				
1	11,081 (75.9)	5,999 (32.0)	597 (49.1)	3,046 (23.6)
2	2,051 (14.0)	3,767 (20.0)	184 (15.1)	2,448 (18.9)
*≥* 3	1,474 (10.1)	9,005 (48.0)	436 (35.8)	7,434 (57.5)
**ISS** ^**1**^			N/A^2^	N/A^2^
1	13,385 (91.7)	5,802 (31.0%)		
*≥* 2	1,217 (8.3)	12,928 (69.0)		
**ICU (days)**				
*≥* 1	51 (0.4)	1,100 (5.9)	45 (3.7)	1,052 (8.1)
**Surgical intervention**				
Underwent surgery	342 (2.3)	7,054 (37.6)	276 (22.7)	6,275 (48.5)
**Mortality** ^**3**^	3(0.02)	115 (0.6)	3(0.3)	115 (0.9)

P<0.0001

Length of Stay – LOS; Injury Severity Score – ISS; Intensive Care Unit - ICU

^1^Unknown ISS n=45

^2^N/A = variable not relevant for this group

^3^The comparison of this variable among patients classified in the ISS >2 group was not statistically significant (p=0.7931)

**Table 3:** Injury and hospitalization characteristics among the pregnant women by gestational age 2012–2021

	First trimester *n* (%) (*n* = 273)	Second trimester *n* (%) (*n* = 2,816)	Third trimester *n* (%) (*n* = 5,712)	Unknown gestational age *n* (%) (*n* = 5,805)
**Mechanism of injury**				
Traffic	148 (54.2%)	1,568 (55.7%)	2,661 (46.6%)	3,186 (54.9%)
Falls	76 (27.8%)	1,002 (35.6%)	2,646 (46.3%)	2,164 (37.3%)
Intentional	25 (9.2%)	95 (3.4%)	81 (1.4%)	127 (2.2%)
Other/unknown	24 (8.8%)	151 (5.4%)	324 (5.7%)	328 (5.7%)
**LOS (days)**				
1	146 (53.5%)	2,166 (76.9%)	4,357 (76.3%)	4,412 (76.0%)
*≥* 2	127 (46.5%)	650 (23.1%)	1,355 (23.7%)	1,393 (24.0%)
**ISS** ^**1**^				
1	174 (64.0%)	2,551 (90.6%)	5,230 (91.6%)	5,430 (93.6%)
*≥* 2	98 (36.0%)	265 (9.4%)	481 (8.4%)	373 (6.4%)
**Injured body region** ^2^				
Head and Neck	77 (28.2%)	246 (8.7%)	330 (5.8%)	348 (6.0%)
Torso	176 (64.5%)	2,293 (81.4%)	4,433 (77.6%)	4,348 (74.9%)
Extremities	101 (37.0%)	442 (15.7%)	881 (15.4%)	607 (10.5%)

P<0.0001

Injury Severity Score – ISS; Length of Stay – LOS

^1^Missing data for ISS: first trimester n=1, third trimester n=1, unknown gestational age n=2

^2^A casualty may be injured in more than one body region

## Data Availability

The datasets analyzed during the current study are available from the corresponding author on reasonable request.

## Abbreviations

ATLS: Advanced Trauma Life Support
EMS: Emergency medical services
ER: Department of emergency medicine
ICU: Intensive care unit
INTR: Israel National Trauma Registry
IQR: Interquartile range
IRB: Institutional Review Board
ISS: Injury Severity Score
LOS: Length of stay
SMC: Sheba Medical Center
TC: Trauma center

## References

[1] Liggett MR, Amro A, Son M, Schwulst S. Management of the pregnant trauma patient: a systematic literature review. J Surg Res. 2023;285:187–96. 36689816 10.1016/j.jss.2022.11.075

[2] Lucia A, Dantoni SE. Trauma management of the pregnant patient. Crit Care Clin. 2016;32(1):109–17. 26600448 10.1016/j.ccc.2015.08.008

[3] Greco PS, Day LJ, Pearlman MD. Guidance for evaluation and management of blunt abdominal trauma in pregnancy. Obstet Gynecol. 2019;134(6):1343–57. 31764749 10.1097/AOG.0000000000003585

[4] Petrone P, Jiménez-Morillas P, Axelrad A, Marini CP. Traumatic injuries to the pregnant patient: a critical literature review. Eur J Trauma Emerg Surg. 2019;45(3):383–92. 28916875 10.1007/s00068-017-0839-x

[5] Melamed N, Aviram A, Silver M, Peled Y, Wiznitzer A, Glezerman M, et al. Pregnancy course and outcome following blunt trauma. The Journal of Maternal-Fetal & Neonatal Medicine. 2012;25(9):1612–7. 22191714 10.3109/14767058.2011.648243

[6] Murphy NJQJD. Trauma in pregnancy: assessment, management, and prevention. Am Fam Physician. 2014;90(10):717–22. 25403036

[7] Deshpande NA, Kucirka LM, Smith RN, Oxford CM. Pregnant trauma victims experience nearly 2-fold higher mortality compared to their nonpregnant counterparts. Am J Obstet Gynecol. 2017;217(5):590.e1-590.e9. 28844826 10.1016/j.ajog.2017.08.004

[8] Mendez-Figueroa H, Dahlke JD, Vrees RA, Rouse DJ. Trauma in pregnancy: an updated systematic review. Am J Obstet Gynecol. 2013;209(1):1–10. 23333541 10.1016/j.ajog.2013.01.021

[9] Makino Y, Kiguchi T, Kato H, Inada S. Epidemiology and outcomes of pregnant trauma patients in japan: a nationwide descriptive study. Eur J Trauma Emerg Surg. 2023;49(3):1287–93. 36385207 10.1007/s00068-022-02165-w

[10] American College of Surgeons. Committee on Trauma. ATLS, Advanced Trauma Life Support for Doctors: Student Course Manual. In: ATLS, Advanced Trauma Life Support for Doctors: Student Course Manual. 9th ed. 2012.

[11] Sato N, Cameron P, Thomson BN, Read D, McLellan S, Woodward A, et al. Epidemiology of pregnant patients with major trauma in Victoria. Emerg Med Australas. 2022;34(1):24–8. 34164928 10.1111/1742-6723.13816

[12] Al-Thani H, El-Menyar A, Sathian B, Mekkodathil A, Thomas S, Mollazehi M, et al. Blunt traumatic injury during pregnancy: a descriptive analysis from a level 1 trauma center. Eur J Trauma Emerg Surg. 2019;45(3):393–401. 29589039 10.1007/s00068-018-0948-1

[13] Battaloglu E, McDonnell D, Chu J, Lecky F, Porter SK. Epidemiology and outcomes of pregnancy and obstetric complications in trauma in the United Kingdom. Injury. 2016;47(1):184–7. 26404664 10.1016/j.injury.2015.08.026

[14] Miller N, Biron-Shental T, Peleg K, Fishman A, Olsha O, Givon A et al. Are pregnant women safer in motor vehicle accidents? J Perinat Med. 2016;44(3):329–32. 10.1515/jpm-2015-0163 26356252

[15] Azar T, Longo C, Oddy L, Abenhaim HA. Motor vehicle collision-related accidents in pregnancy. J Obstet Gynaecol Res. 2015;41(9):1370–6. 26179944 10.1111/jog.12745

[16] Vivian-Taylor J, Roberts C, Chen J, Ford J. Motor vehicle accidents during pregnancy: a population‐based study. BJOG. 2012;119(4):499–503. 22324920 10.1111/j.1471-0528.2011.03226.x

[17] Maxwell BG, Greenlaw A, Smith WJ, Barbosa RR, Ropp KM, Lundeberg MR. Pregnant trauma patients may be at increased risk of mortality compared to nonpregnant women of reproductive age: trends and outcomes over 10 years at a level I trauma center. Womens Health. 2020;16:174550652093302. 10.1177/1745506520933021 PMC7315661 32578516

[18] Owattanapanich N, Lewis MR, Benjamin ER, Wong MD, Demetriades D. Motor vehicle crashes in pregnancy: maternal and fetal outcomes. J Trauma Acute Care Surg. 2021;90(5):861–5. 33496550 10.1097/TA.0000000000003093

[19] Cahill AG, Bastek JA, Stamilio DM, Odibo AO, Stevens E, Macones GA. Minor trauma in pregnancy—is the evaluation unwarranted? Am J Obstet Gynecol. 2008 Feb;198(2):208.e1-208.e5. 10.1016/j.ajog.2007.07.042 18226625

[20] Pearlman MD, Tintanalli JF, Lorenz RP. A prospective controlled study of outcome after trauma during pregnancy. AM JObstet Gynecol. 1990;162:1502–10. 2360584 10.1016/0002-9378(90)90913-r

[21] Jain V, Chari R, Maslovitz S, Farine D, Bujold E, Gagnon R, et al. Guidelines for the management of a pregnant trauma patient. J Obstet Gynaecol Can. 2015;37(6):553–71. 26334607 10.1016/s1701-2163(15)30232-2

